# Validation of an instrument to evaluate health promotion at schools

**DOI:** 10.1590/S01518-8787.2016050005855

**Published:** 2016-02-19

**Authors:** Raquel Oliveira Pinto, Marcos Pascoal Pattussi, Larissa do Prado Fontoura, Simone Poletto, Valenca Lemes Grapiglia, Alexandre Didó Balbinot, Vanessa Andina Teixeira, Rogério Lessa Horta

**Affiliations:** I Programa de Pós-Graduação em Saúde Coletiva. Universidade do Vale do Rio dos Sinos. São Leopoldo, RS, Brasil; II Programa de Pós-Graduação em Saúde Coletiva. Unidade de Pesquisa e Pós-Graduação. Universidade do Vale do Rio dos Sinos. São Leopoldo, RS, Brasil

**Keywords:** School Health, Health Promotion, Program Evaluation, Evaluation of Research Programs and Tools, Reproducibility of Results, Validation Studies

## Abstract

**OBJECTIVE:**

To validate an instrument designed to assess health promotion in the school environment.

**METHODS:**

A questionnaire, based on guidelines from the World Health Organization and in line with the Brazilian school health context, was developed to validate the research instrument. There were 60 items in the instrument that included 40 questions for the school manager and 20 items with direct observations made by the interviewer. The items’ content validation was performed using the Delphi technique, with the instrument being applied in 53 schools from two medium-sized cities in the South region of Brazil. Reliability (Cronbach’s alpha and split-half) and validity (principal component analysis) analyses were performed.

**RESULTS:**

The final instrument remained composed of 28 items, distributed into three dimensions: pedagogical, structural and relational. The resulting components showed good factorial loads (> 0.4) and acceptable reliability (> 0.6) for most items. The pedagogical dimension identifies educational activities regarding drugs and sexuality, violence and prejudice, auto care and peace and quality of life. The structural dimension is comprised of access, sanitary structure, and conservation and equipment. The relational dimension includes relationships within the school and with the community.

**CONCLUSIONS:**

The proposed instrument presents satisfactory validity and reliability values, which include aspects relevant to promote health in schools. Its use allows the description of the health promotion conditions to which students from each educational institution are exposed. Because this instrument includes items directly observed by the investigator, it should only be used during periods when there are full and regular activities at the school in question.

## INTRODUCTION

Since the 1980s, which saw the signing of the Ottawa Charter for health promotion, schools have been seen as suitable environments for promoting health. This theme has been further expanded on several fronts, such as on the Jakarta Declaration (1997), in the creation of the European Network of Health Promoting Schools^29^ and on initiatives involving all continents^2^ ^,^ ^17^ ^,^ ^26^ ^,^ ^27^. To strengthen disease prevention and health promotion in Brazilian schools, the Brazilian Government instituted the *Programa Saúde na Escola* (PSE – School Health Program)^19^, along with actions to promote healthy eating, a culture of peace, human rights, reduced consumption of alcohol and other drugs and sexual and reproductive health, as well as anthropometric, oral and visual evaluations and periodical vaccinations.

These initiatives have been recognized as beneficial, but the instruments to assess them have been debated^18^ ^,^ ^28^. The available evaluations are specific, for nutritional health^5^ ^,^ ^9^, mental health^27^, substance use^2^ ^,^ ^22^ or physical activity^14^. Only a small number of studies measure environmental conditions^4^. Thus, more comprehensive options to evaluate health-promoting practices in school have emerged, which include using the schools’ environments and their conditions as an element for analysis, while using various forms of data collection and adapting the experiences of others to the needs and characteristics of their locations or regions^15^ ^,^ ^18^ ^,^ ^26^ ^,^ ^27^. The validation of these instruments is still incipient and limited^18^ ^,^ ^28^.

The objective of this article was to validate the instrument whose purpose is to evaluate health promotion in the school environment.

## METHODS

To select the items that would be included in the questionnaire, we reviewed the literature in the following databases: Web of Science, PubMed, SciELO, EBSCO Information Services, Psycoinfo, Educational Resources Information Center (Eric), Eric Proquest, Science Direct and the Virtual Health Library (VHL), using *evaluation* AND *health promotion* AND *school environment* as descriptors. We selected empirical articles and literature reviews, published between April 2004 and April 2013, which considered the school environment as a study variable, as well as documents from the public domain such as public policy protocols and international guidelines. The following were excluded: clinical trials and observational studies whose study populations were schools but did not evaluate the school context; articles written in languages other than English, Spanish, Portuguese or French; and publications that did not address the topic of interest. The databases were consulted in 2013, and we read the abstracts to determine whether they would be read in full and whether they would be included in the study or not. All the selected articles were read in two steps: recognition of the design and confirmation of inclusion; and selection of the items regarding health promotion in school to be included in the instrument. We found 436 empirical articles or reviews, of which 353 were excluded (243 for not addressing the topic in question, 81 for being publications that only used schoolchildren as the study population, four for not being available in the aforementioned languages and 25 for being duplicates). From the total selected, after reviewing and reading all their abstracts, 83 articles were read in full. In addition, 14 documents from the public domain were used (ordinances and resolutions from International Bodies, Education or Health Ministries, the Public Ministry and State Health or Education Secretariats).

For the purposes of analyzing the selected articles and other documents, 40 questioning items directed towards the school’s manager (questioned) and 20 items obtained through direct observation (observed) were created. The 60 items were grouped into three dimensions, according to their main characteristic: pedagogical, structural and relational (Tables 1 to 5). The pedagogical dimension was made up of 14 items and looked at themes and activities related to the learning process, from a perspective of attaining healthy environments. Items considered relevant and feasible to be analyzed individually or cross-sectionally were: healthy eating and physical activity^14^, personal hygiene care^26^ ^,^ ^27^, sexual and reproductive health^8^; prevention of licit and illicit drug use^2^ ^,^ ^19^ ^,^ ^22^; culture of peace and human rights and personal interaction skills in terms of inclusion, respect, initiative and tolerance^16^ ^,^ ^19^ ^,^ ^25^.

**Table 1:** Pedagogical dimension* with items, commonalities and factorial loads of the items from the questionnaire to evaluate health promotion in the school.

Theme	Item	h^2^	Factors			

			1	2	3	4
Drugs and sexuality						
	(Questioned) Educational activities at school that stimulate debate regarding the risks associated with the consumption of:					
	alcoholic beverages	0.837	0.874	-	-	-
	tobacco (cigarettes, cigars)	0.867	0.868	-	-	-
	illicit drugs	0.904	0.858	0.393	-	-
	(Questioned) Educational activities that promote debate on sexual health, reproductive health and STDs (sexually transmitted diseases).	0.594	0.698	-	0.307	-
Violence and prejudice						
	(Questioned) Educational activities to stimulate reflection and discussion on:					
	bullying (hostility, coercion, constraint)	0.789	0.358	0.792	-	-
	discrimination and prejudice	0.784	0.382	0.751	-	-
	sexual diversity/homophobia	0.589	-	0.728	-	-
Self-care						
	(Questioned) Educational activities regarding healthy eating in different areas of the school.	0.783	-	-	0.856	-
	(Questioned) Educational activities relating to personal skills such as empathy, interpersonal relations, decision-making, critical and creative thinking, pressure and/or stress management, self-understanding.	0.781	-	-	0.848	-
	(Questioned) Educational activities that address and stimulate personal hygiene at school.	0.709	-	-	0.713	0.437
Peace and quality of life						
	(Questioned) Educational activities on a culture of peace and human rights.	0.698	-	-	0.338	0.739
	(Questioned) Educational activities relating to physical exercise at school, not considering those that are part of the Physical Education curriculum (e.g., games, contests, dances, bouts, running, gymnastics, team sports or other).	0.551	-	-	-	0.728
	(Questioned) Educational activities to stimulate reflection and discussion on violence (domestic, sexual and other).	0.728	-	0.54	-	0.657
General Cronbach's alpha of the dimension	0.843	Alpha	0.89	0.74	0.79	0.61
Split-half coefficient	0.856	Variance %	37.2	16.9	10.5	9.4
KMO	0.705	Cumulative %	37.2	54.1	64.5	73.9
Bartlett	< 0.001	-	-	-	-	-

KMO: Kaiser-Meyer-Olkin test; Bartlett: Bartlett’s sphericity test; h^2^: commonality

* The items in this dimension are preceded by orientation: Answer if your school continuously and permanently develops the actions/programs presented below: (Projects in development, those not yet implemented, or those run for a period, but subsequently stopped, should not be considered!)

**Table 5:** Question excluded in the adjustments following the reliability or principal components analyses.

Dimension	Questions
Structural	(Questioned) Do you consider that the Parent-Teacher Council at your school is an effective body?
	(Questioned) Does the school have any permanent project in which students have the opportunity to participate in educational/recreational activities outside the school environment?
	(Questioned) Does the school open up its space or offer access to the local community for educational and leisure activities at weekends? (e.g., open school)
	Answer if your school continuously and permanently develops the actions/programs presented below: (Projects in development, those not yet implemented, or those run for a period, but subsequently stopped, should not be considered!)
	(Questioned) Does the school have any partnerships with institutions/technical support from health professionals on health guidance in general.
	(Questioned) Are there appropriate facilities to provide a healthy diet (own dining hall with adequate space and structure)?
	(Questioned) Does the school develop sanitary practices to prevent the spread of disease at school and in the community such as controlling vectors such as rats and insects, and properly disposing garbage? (consider the whole school).
	Does the school have structural conditions that are compatible with:
	(Questioned) fire prevention (license issued by the local authority)
	Do you consider the physical structure of the classrooms adequate in terms of:
	(Questioned) accident prevention?
	(Questioned) climate control (temperature and humidity)?
	(Observed) Access to foods with a high content of fat, saturated fat, trans fat, sugar and salt (e.g., biscuits with fondant filling, fried foods, sweets in general, savory snacks).
	(Observed) Cafeteria properly structured in general terms of cleanliness and organization.
	(Observed) (Observed) Posters, flyers or any other form of information access for individuals frequenting the school regarding smoking, alcohol and drugs in general.
	(Observed) Presence of security cameras in internal or external common areas.
	(Observed) Recycle bins for proper disposal, with separation facilities for dry and organic waste.
	(Observed) Sports court or area for performing sports or physical activity, attached to the school, with covered and outdoor space.
	(Observed) Maximum of up to 35 students per class (5^th^ to 8^th^ grade).
Relational	(Questioned) Does the school have student body or any other social group in which all students have the opportunity to participate in the school’s decision-making process?
	(Questioned) Have there been any incidents of verbal abuse in the school environment between students and students over the last 30 school days?
	(Questioned) Have there been any physical conflicts between students and students in the school environment over the last 30 schooldays?
	(Questioned) Have there been any security-related issues involving weapons (firearms or blade weapons), theft and vandalism, regardless of whether or not police/municipal guards/security officers had been called, in the school environment over the last 30 schooldays?

No item was excluded from the pedagogical dimension.

The structural dimension included 33 items. We considered resources in terms of physical and installed capacity, suitability of space for educational activities^11^ ^,^ ^14^, and appropriateness of personal safety and sanitary conditions^17^. We also included items regarding the relationship with the surrounding community and school partnerships that make acquiring resources for health promotion and disease prevention possible^13^ ^,^ ^14^.

The relational dimension, with 13 items, linked the conditions that were considered necessary to construct an ethos to promote a pleasant environment from a social point of view, which focused on the relationships and the conditions that had been established within the school community. Aspects regarding the relationship between students, teachers and the community, the presence of violence, actions to promote protagonism and respect for rules of coexistence were also considered^3^ ^,^ ^19^ ^,^ ^27^ ^,^ ^29^.

Each of the 60 items was designed in the form of a question, which corresponded to dichotomous categorical variables (yes; no). As some items involve direct observation, the use of the instrument requires that the person applying it visit the school and verify whether or not the proposed condition exists.

The first proposed version was submitted to content validation using the Delphi method^10^ ^,^ ^12^ ^,^ ^24^ *.* We invited five specialists in the areas of education and health, contacted by electronic means or in person. All were requested to sign a form of free and informed consent. All received the instrument so they could give their opinions as to whether each question and guidance item was properly designed (yes; no). The aim of this procedure was to survey the understandability of the questionnaire, so that the respondents could support their answers through justifications and suggestions to improve the text. After the first evaluation by means of the Delphi method, among the 61 sent questions (60 items from the instrument and one instruction), the agreement among the evaluators was greater than 70.0% for all the items. None of the questions were dismissed and a second evaluation was performed with only seven questions receiving suggestions or criticism. A 100% agreement, was, then, achieved, which meant that a third evaluation was not required.

The redesigned instrument was presented to 55 school managers from two medium-sized cities (of around 70,000 inhabitants) in the South region of Brazil. It was requested that the schools’ director or another member of the management team participate by completing the questionnaire. Each institution defined who would be its responding representative. Two private schools from the same city refused to participate in the research. We visited 31 schools in the first city and 22 in the second, totaling 53 interviews. The questionnaires were applied by three researchers following their training and after they had applied a pilot study in four schools from a different city with similar characteristics.

The data were inputted into SPSS 20.0 statistical software and submitted to descriptive analysis. Some items of the questionnaire were excluded during the successive analysis stages according to the following criteria: questions with answers from, at most, two schools (among the 53 visited) in any of its categories, because the low frequency hindered its belonging to any construct; items that, with their exclusion, increased the internal consistency of the scale and items with weak loads (< 0.3) on more than one factor (not belonging to any factor).

Each dimension proposed in the literature was subjected to factorial analysis using principal component analysis. The adequacy of the data from the sample was evaluated using the Kaiser-Meyer-Olkin (KMO) test, whose recommended value must exceed 0.6, and the Bartlett’s sphericity test in which a specific statistical significance should be reached (p < 0.05). The number of factors to be extracted from each dimension was defined using open criteria, i.e., only those with eigenvalues above 1, done to identify the factors that contribute to the variance in the original variables. To minimize the number of variables with high loads in each factor, we used the Varimax rotation which obtained the representatives components from the instrument’s underlying dimensions. Values greater than or equal to 0.3 for the values of the commonalities were considered acceptable, which are understood as the proportions of the variances for each variable included in the analysis and are explained by the extracted components.

To ensure that each item corresponded to the construct’s underlying structure, a factorial load criteria equal to or greater than 0.4 was considered so that the item belonged to the construct. Despite the indication that items with a factorial load higher than one factor were excluded^7^, we allocated it where the item would contribute more theoretically and significantly to the final cluster of a factor.

We used Cronbach’s alpha to evaluate internal consistency, which evaluates how well a cluster of items unidimensionally measures the latent construct proposed by the scale. Values equal to or greater than 0.6 were considered acceptable. The split-half reliability was also tested, which is a measure of consistency in which the sample is divided into two and the test scores for each half are compared with each other. If the results are similar, it is believed that the same construct is being measured in the two halves. Minimum values of 0.6 are acceptable for each dimension^1^ ^.^

This study was approved by the Research Ethics Committee at the Universidade do Vale do Rio dos Sinos (Process 025/2013). Only after making contact with the municipal and state bodies responsible for the schools were their respective managers invited to participate. The interviews were only performed after the free and informed consent forms were read and signed. The schools’ identifications were recorded in the instrument, with the confidentiality of data being maintained.

## RESULTS

Twenty-eight questions remained on the instrument after the construct’s validation analysis. Tables 1, 2 and 3 show the results of the principal components analysis and the internal consistency conducted for each of the dimensions proposed by the literature.

**Table 2:** Structural dimension with items, commonalities and factorial loads of the items from the questionnaire to evaluate health promotion in the school.

Theme	Item	h^2^	Factors		

			1	2	3
Access					
	(Questioned) Does the school have structural conditions that are compatible with accessibility (physical environment that enables students with special needs to access the same educational activities that are provided to others)?	0.820	0.898	-	-
	(Observed) Accessibility (physical environment which permits persons with disabilities access to all activities, such as ramps, floors and rooms that are suitable for wheelchairs)?	0.775	0.805	-	0.334
	(Observed) Access to the only point leading to the inside of the school (or similar) permanently monitored by porter or security guard.	0.44	0.626	-	-
Structure conservation and equipment					
	(Observed) Evidence of problems to conserve the structure, such as broken chairs being used, holes, leaks, broken tiles, risk of falling due to floor conditions or other.	0.674	-	0.819	-
	(Observed) Library occupying an exclusive room, reading tables, chairs, bookshelves, windows with protection from the sun, and a minimum of seats equivalent to at least 50.0% of the school’s students who attend the study time with the most students.	0.658	-	0.786	-
	(Questioned) Does the school have structural conditions that are consistent with environmental preservation (sustainable energy use, tree planting, recycling)?	0.508	-	0.505	0.414
Sanitary conditions					
	(Observed) Covered and outdoor physical space/recreation area in conditions suitable for recreational activities, not counting the areas reserved for sports, with an area equivalent to at least 1/3 of the total area occupied by classrooms (not including the common areas).	0.698	-	-	0.818
	(Questioned) Does the school have its own health team or the support of a local health service team that performs periodic health evaluations and provides guidance to its students?	0.596	-	-	0.726
	(Observed) Bathrooms in working order, properly preserved equipment (clean flushable toilets with adequate water, access to sinks for washing hands and general cleaning) and brushodrome or suitable structures to allow children, including the smallest ones, to brush their teeth.	0.552	-	0.508	0.519
General Cronbach’s alpha of the dimension	0.737	Alpha	0.72	0.63	0.58
Split-half coefficient	0.805	Variance %	33.1	16.1	14.4
KMO	0.639	Cumulative %	33.1	49.2	63.5
Bartlett	< 0.001	-	-	-	-

KMO: Kaiser-Meyer-Olkin test; Bartlett: Bartlett’s sphericity test; h2: commonality

**Table 3:** Relational dimension with with items, commonalities and factorial loads of the items from the questionnaire to evaluate health promotion in the school.

Theme	Items	h^2^	Factors	

			1	2
Community relations				
	(Observed) Generally, the school’s surrounding area can be considered pleasant and suitable for children and adolescents to frequent.	0.634	0.794	-
	(Observed) Evidence of physical damage to the school, such as graffiti, vandalism or other signs of vandalism against the property.	0.584	0.754	-
	(Questioned) Does your school participate in organizations or have any partnerships of interest with the local community, involving councils, authorities, NGO’s, local leaders, social or any other groups?	0.405	0.589	-
Relationships at school				
	(Questioned) in the last 30 days there have been episodes of school fights/arguments between people from the local community and school representatives?	0.756	-	0.869
	(Questioned) Have there been any incidents of verbal abuse in the school environment between teachers and teachers over the last 30 school days?	0.529	0.402	0.606
	(Questioned) Have there been any incidents of verbal abuse in the school environment between students and teachers over the last 30 school days?	0.339	-	0.613
General Cronbach's Alpha of the dimension	0.642	Alpha	0.58	0.49
Split-half coefficient	0.642	Variance %	38.1	17.1
KMO	0.681	Cumulative %	38.1	55.1
Bartlett	< 0.001	-	-	-

KMO: Kaiser-Meyer-Olkin test; Bartlett: Bartlett’s sphericity test; h2: commonality

The requirements for factor analysis were achieved with a KMO above 0.6 in the three dimensions and a statistically significant Bartlett’s sphericity test (p < 0.001). The commonalities for most of the items also reached desirable values close to or greater than 0.4. During the analysis of internal consistency for dimensions and factors, the majority of the analyses showed satisfactory results for a homogeneous construct (above 0.6).

Table 1 presents the pedagogical dimension, which maintained a total of 13 items. The KMO was equal to 0.705 and Bartlett’s sphericity reached statistical significance (p < 0.001). The principal component analysis showed four components: drugs and sexuality (explaining 37.2% of the variance)*;* violence and prejudice (16.9% of the variance)*;* self-care (10.5% of the variance); peace and quality of life (9.4% of the variance), all with eigenvalues greater than 1. The split-half test value was 0.856 for the dimension. Cronbach’s Alpha was above 0.7 for the extracted factors, except for the component named peace and quality of life, which had a value of 0.61. The commonality (h^2^) only produced values above the recommended minimum of 0.3.

Regarding the structural dimension (Table 2), the KMO was equal to 0.639 and the Bartlett’s test of sphericity reached statistical significance (p < 0.001). From the principal component analysis, there were three constant components: access, conservation, and equipment and sanitary structure, with eigenvalues above 1, which explains the 33.1%, 16.1% and 14.4% of the variance, respectively. The value in the split-half test for this dimension was 0.805. Cronbach’s alpha was above 0.7 in the component named access*,* with values close to 0.6 in the other two components (0.63 and 0.58). The commonality (h^2^) only produced values above the recommended minimum of 0.3.

In the relational dimension (Table 3), the KMO was equal to 0.681 and the Bartlett’s test of sphericity reached statistical significance (p < 0.001). During the principal component analysis, the items were grouped into: community relations (explaining 38.1% of variance) and relationships at school (explaining 17.1% of the variance), all with eigenvalues greater than 1. The value in the split-half test was 0.646 in this dimension. Cronbach’s alpha was below 0.7 in both factors. The commonality (h^2^) only produced values above the recommended minimum of 0.3.

We excluded 12 items in the frequency analysis of the responses (Table 4) and 20 in the analysis of principal components and reliability, according to the exclusion criteria employed (Table 5).

**Table 4:** Questions excluded, following the frequency analyses, for receiving a maximum two registries in at least one of the response categories.

Dimension	Questions
Pedagogical	Answer if your school continuously and permanently develops the actions/programs presented below: (Projects in development, those not yet implemented, or those run for a period, but subsequently stopped, should not be considered!):
	(Questioned) Are school meals offered and/or food and healthy meals (e.g., fruits, natural juices, snacks or meals low in sugar, salt and fats) for sale at school?
Structural	(Questioned) Are the bathrooms connected to the sewage network.
	Do you consider the physical structure of the classrooms adequate in terms of:
	(Questioned) Natural lighting (including protection against direct sunlight)?
	(Questioned) Ventilation?
	(Observed) Access to food and healthy meals (e.g., fruits, natural juices, snacks or meals low in sugar, salt and fats).
	(Observed) Posters, flyers or any other form of information access for individuals frequenting the school regarding sexual and reproductive health.
	(Observed) Ventilated rooms, with adequate and direct aeration.
	(Observed) Adequate lighting in classrooms along with protection against direct sunlight.
	(Observed) Minimum of 1.20 m^2^ of space per pupil in each room (mean room size in m^2^/mean number of students per room).
Relational	(Observed) Does the school have rules (clearly defined standards) regarding rights and duties at school?
	(Observed) Have there been any physical conflicts between students and teachers in the school environment over the last 30 schooldays
	(Observed) Have there been any physical conflicts between teachers and teachers in the school environment over the last 30 schooldays?

## DISCUSSION

The evaluated instrument includes improvements compared to existing ones: it has a reduced dimension, with easy applicability; the items are all presented in the form of questions with “yes or no” answers, which avoids ambiguities regarding whether or not that feature or process exists; it is the first that includes items that have been directly observed by whomever collected the data, which reduces the bias from managers who have certain ideals about their own school; it was designed to be applied only once in each school, without being proposed to different members of the school community, which avoids the information collected reflecting the ideological perspectives or oscillating between dissatisfaction and satisfaction with the school, which can vary widely in the same community; and work issues such as sexuality and violence, which are part of daily life for communities in developing countries, but are not included or are not featured in other instruments.

The instrument’s final composition can be considered appropriate since, in addition to the satisfactory values from the overall reliability and validity analyses, the dimensions are composed of items that coalesce in components that are compatible with themes indicated as priorities for health promotion in the school environment. These are: drugs^2^ ^,^ ^3^ ^,^ ^20^ ^,^ ^22^, sexuality^4^ ^,^ ^8^ ^,^ ^13^ ^,^ ^21^, violence^19^ ^,^ ^25^, prejudice^19^ ^,^ ^25^, self-care^26^ ^,^ ^27^ ^,^ ^29^, culture of peace^19^ ^,^ ^25^ ^,^ ^27^, quality of life^15^ ^,^ ^18^ ^,^ ^26^ ^,^ ^27^, accessibility and security^19^ ^,^ ^25^ ^,^ ^27^, sanitary conditions^15^ ^-^ ^18^, conservation of the structure and equipment available to the school community^11^ ^,^ ^14^ ^,^ ^18^ ^,^ ^27^, in addition to relationships within the school and with the community^3^ ^,^ ^4^ ^,^ ^18^ ^,^ ^24^.

In addition to the efforts made by the World Health Organization, who proposed a broad instrument to investigate health in schools (the Global School-based Student Health Survey [GSHS])^22^, the Center for Disease Control in the United States has also developed their own instrument^26^. Research initiatives in other countries have also led to the creation of instruments with this purpose^2^ ^,^ ^16^ ^,^ ^18^. In Brazil, in 2012, the *Pesquisa Nacional de Saúde do Escolar* (PeNSE – National Survey of School Health) ^a^ included items regarding the school environment in its questionnaire, to be proposed to the managers of the visited institutions.

Like the instrument featured in this article, the set of items in PeNSE did not employ the Likert scale and the two models are not similar concerning the formulation of various items. No direct observation items were included, which is different from what was employed in some studies on health at school, mainly for observing structural resources, equipment and physical area on the school premises^19^ ^,^ ^29^.

All the instruments developed to date^1^ ^7^ ^,^ ^18^ ^,^ ^22^ ^,^ ^26^ ^,^ ^27^ are central to the principles of the Ottawa Charter for health promotion: health policies at school and personal skills in health, which resemble the pedagogical dimension of the instrument from this study; physical environment and provision of health services, which resemble the structural dimension; social environment; and school and community relations, which resemble the relational dimension. The origin and insertion of the researchers in different national contexts lead instruments to express specific characteristics, which are reflected as differences in the cluster of its items. For example, sexual health is present in this and in an Australian study^27^, while the Korean instrument^18^ analyzes protective measures against disasters and extreme weather situations, which are of little use in some contexts.

The GSHS questionnaire has a very extensive number of items regarding agreed rules in the school environment and investigates these items requesting that its terms are explicit in written form, which may not be customary in some cultures. For example, details of internal regulations included within public documents are not common in Brazilian schools.

Another difference might also be perceived in the security item, since the transit of vehicles within the school environment was a concern in countries with lower rates of urban violence^18^ ^,^ ^26^ ^,^ ^27^, while for the Brazilian reality, access to school and the school environment seemed more relevant^19^. In Brazil, violence represents a social problem and the school is seen as a suitable setting to help deal with this situation, which requires both educational and public security interventions^19^ ^,^ ^25^.

The overall analysis of the pedagogical dimension suggested a good level of acceptable validity and reliability, with the highest factorial load being in the drugs and sexuality component. The explicit dimension was satisfactorily in line with health promotion in school, prioritizing actions that give schools the opportunity to make healthy choices and encourage self-care.

The sexual health theme presented a good factorial load, but was excluded due to its representation in the structural dimension, where it appeared as a direct observation of the existence of informational materials on gender and sexuality in the common areas of the school. The theme had already been excluded in frequency analysis, as it was absent in 52 of the 53 schools visited. This, which is apparently contradictory to the load of the theme in the pedagogical dimension, is suggestive of the difficulty, high even today, of information related to sexuality being accessible in the school context–with the subject still a matter that is avoided. Without a more explicit proposal of information, other than as a classroom activity (thus, of a eventual and collective nature), access to information and some kind of individual support, while preserving the intimacy of those looking for it, appears to be of limited scope^8^ ^,^ ^13^ ^,^ ^21^.

During the overall analysis of the structural dimension, a good level of validity and reliability was considered acceptable. The component regarding the sanitary structure presented a Cronbach’s alpha slightly below what is deemed acceptable, however, it was made up of essential items^2^ ^,^ ^11^ ^,^ ^14^ ^,^ ^26^ and its preservation reinforced the overall evaluation of the dimension. This dimension’s items had good factorials loads, with better results in the access component, which encompassed relevant aspects in terms of structural features of accessibility and safety in the school environment, which is also in agreement with the literature^2^ ^,^ ^16^ ^-^ ^19^ ^,^ ^27^.

During the general analysis of the relational dimension, we observed good validity and acceptable reliability. We found adequate factorial loads in the constituent items of each component, showing the school’s social climate^19^ ^,^ ^24^. The students’ participation item in the processes of picking the school was excluded from this study’s validity and reliability analysis and represented a considerable and important loss of information^15^. This fact may have been due to the sample size used, which was smaller than what is recommended for validation analysis. The number of five subjects per item has been suggested^23^. An instrument of 60 items should be tested in approximately 300 schools. It is possible that increasing the number of schools, relevant items that did not remain in this case, would be included, or even a greater number of factors could be found. This also has an impact on the reliability analyses, since scales with few items tend to provide lower Cronbach’s alpha results^6^.

The limitation represented by the low number of schools where the instrument was applied might also explain, at least in part, why the instrument did not have an excellent performance, but rather only satisfactory with good factorials loads (> 0.4) and acceptable reliability (> 0.6). The Korean instrument^18^ was designed to be answered by members of the school community, unlike the one from this study, which has a singular application. When only a single data collection exist per school by a properly trained investigator, with items from direct observation, more objective data tends to be generated. The seven factors of that instrument had Cronbach’s alphas ranging from 0.86 and 0.97 for the instrument’s cluster. The Korean instrument was the first to be proposed and subjected to reliability and validity analyses, which includes a confirmatory factor analysis. The other instruments are not able to provide sufficient assurance for validity and reliability^18^. The Australian instrument^2^ ^,^ ^27^, for example, is referred to as having had good performance in the overall reliability analysis, with a Cronbach’s alpha result of 0.88, but without reaching consistent factorial structures so as to employ factor analysis and validate the construct^18^.

Another limiting factor of this study was that it only evaluated schools in cities located in the Southern region of Brazil, which were similar in terms of population size. Studies with other populations and with greater heterogeneity of school environments would enable the evaluation of the consistency of the results and obtain a better evaluation of the instrument.

The data suggest that the proposed structure is consistent, with a sufficient number of dimensions and factors. This instrument can be applied as it currently exists, with 28 items, but it is recommended that further studies are undertaken, even with 60 original items, so that the local realities of the different regions of Brazil are included, with their distinct social and demographic characteristics. In addition, other psychometric properties such as reproducibility and criteria-related validity still need to be studied in such a way as to complement and further qualify the validation process of the instrument.

## Footnotes

a Ministério do Planejamento, Orçamento e Gestão (BR); Instituto Brasileiro de Geografia e Estatística. Pesquisa nacional de saúde do escolar 2012. Rio de Janeiro (RJ): IBGE; 2013.
