# Supplementary material for: Validation of an instrument to evaluate health promotion at schools
Source: Rev Saude Publica. 2016 Feb 19;50:2. doi: 10.1590/S01518-8787.2016050005855 (PMC4772694; doi:10.1590/S01518-8787.2016050005855)
Supplement: Translation

# Translation: Validação de instrumento desenvolvido para avaliação da promoção de saúde na escola

**Authors:** Raquel Oliveira Pinto, Marcos Pascoal Pattussi, Larissa do Prado Fontoura, Simone Poletto, Valenca Lemes Grapiglia, Alexandre Didó Balbinot, Vanessa Andina Teixeira, Rogério Lessa Horta

**Affiliations:** I Programa de Pós-Graduação em Saúde Coletiva. Universidade do Vale do Rio dos Sinos. São Leopoldo, RS, Brasil; II Programa de Pós-Graduação em Saúde Coletiva. Unidade de Pesquisa e Pós-Graduação. Universidade do Vale do Rio dos Sinos. São Leopoldo, RS, Brasil

**Keywords:** Saúde Escolar, Promoção da Saúde, Avaliação de Programas e Projetos de Saúde, Avaliação de Programas e Instrumentos de Pesquisa, Reprodutibilidade dos Testes, Estudos de Validação

## Abstract

**OBJETIVO:**

Validar instrumento de avaliação da promoção de saúde no ambiente escolar.

**MÉTODOS:**

Para validação do instrumento de pesquisa, foi elaborado questionário com base em diretrizes da Organização Mundial da Saúde, mas adequado ao contexto de saúde escolar no Brasil, com 60 itens, sendo 40 dirigidos aos gestores e 20 para observação direta pelo entrevistador. A validação de conteúdo dos itens foi feita pela técnica Delphi, com aplicação do instrumento em 53 escolas de dois municípios, de porte médio, da região Sul do País. Foram realizadas análises de confiabilidade (alpha de Cronbach e *split-half*) e de validade (análise de componentes principais).

**RESULTADOS:**

O instrumento permaneceu composto por 28 itens, distribuídos em três dimensões: pedagógica, estrutural e relacional. Os componentes resultantes apresentaram boas cargas fatoriais (> 0,4) e confiabilidade aceitável (> 0,6) para a maioria dos itens. A dimensão pedagógica identifica atividades educativas que abordem drogas e sexualidade, violência e preconceitos, autocuidados e paz e qualidade de vida. Estrutura se divide em acesso, estrutura sanitária e conservação e equipamentos. A dimensão relacional inclui relações na escola e relações com a comunidade.

**CONCLUSÕES:**

O instrumento proposto apresenta valores satisfatórios de validade e confiabilidade, englobando aspectos relevantes à promoção de saúde nas escolas. Seu emprego permite retratar condições de promoção de saúde às quais estão expostos os estudantes vinculados a cada instituição de ensino. Como possui itens de observação direta pelo pesquisador, deve ser usado apenas em períodos de atividade plena e regular da escola.

## INTRODUÇÃO

A partir dos anos 1980, com a Carta de Ottawa, escolas são vistas como ambientes promotores de saúde. Isso tem repercutido em diversas frentes, como em Jacarta (1997), na formação da Rede Europeia de Escolas Promotoras de Saúde^29^ e em iniciativas em todos os continentes^2^ ^,^ ^17^ ^,^ ^26^ ^,^ ^27^. No Brasil, para fortalecer a prevenção de doenças e a promoção da saúde na escola, o governo instituiu o Programa Saúde na Escola (PSE)^19^, com ações de promoção de alimentação saudável, cultura de paz e direitos humanos, redução do consumo de álcool e outras drogas e saúde sexual e reprodutiva, além de avaliações antropométricas, visuais, bucais e vacinais periodicamente.

O mérito dessas iniciativas tem sido reconhecido, mas os instrumentos de sua avaliação ainda são discutidos^18^ ^,^ ^28^ ^.^ As avaliações disponíveis são específicas, como para saúde nutricional^5^ ^,^ ^9^, saúde mental^27^, uso de substâncias^2^ ^,^ ^22^ ou atividade física^14^ ^.^ Poucos estudos medem condições ambientais^4^. Surgem, então, opções de avaliação das práticas promotoras de saúde na escola de modo mais abrangente, incluindo o ambiente escolar e suas condições como elemento de análise, utilizando variadas formas de coleta de dados e adaptando as experiências de outros às necessidades e características próprias de suas localidades ou regiões^15^ ^,^ ^18^ ^,^ ^26^ ^,^ ^27^ ^.^ A validação desses instrumentos é, ainda, incipiente e limitada^18^ ^,^ ^28^.

Este artigo tem por objetivo validar instrumento de avaliação da promoção de saúde no ambiente escolar.

## MÉTODOS

Para seleção de itens para compor o questionário, foi realizada revisão da literatura nas bases de dados Web of Science, PubMed, SciELO, EBSCO Information Services, Psycoinfo, Educational Resources Information Center (Eric), Eric Proquest, Science Direct e Biblioteca Virtual em Saúde (BVS), a partir dos descritores *evaluation* AND *health promotion* AND *school environment*. Foram selecionados artigos empíricos e revisões de literatura publicadas entre abril de 2004 e abril de 2013 que considerassem o ambiente escolar como variável de estudo, bem como documentos de domínio público como protocolos de políticas públicas e guias internacionais. Foram excluídos: ensaios clínicos e estudos observacionais cuja população de estudo eram escolares, mas que não avaliavam o contexto escolar; artigos de outros idiomas que não em inglês, espanhol, português ou francês; publicações que não abordavam a temática de interesse. A consulta nas bases de dados foi realizada em 2013, sendo lidos os resumos para determinar sua leitura na íntegra e inclusão ou não no estudo. Todos os artigos selecionados foram lidos em duas etapas: reconhecimento do delineamento e confirmação de inclusão; e seleção dos itens sobre promoção de saúde na escola a serem incluídos no instrumento. Foram encontrados 436 artigos empíricos ou revisões, dos quais 353 foram excluídos, pois 243 não abordavam o tema em questão, 81 eram publicações que apenas possuíam escolares como população de estudo, quatro não estavam disponíveis nos idiomas definidos na revisão e 25 eram duplicatas. Do total selecionado após revisão e leitura dos resumos, 83 artigos foram lidos na íntegra. Foram aproveitados também 14 documentos de domínio público (portarias e resoluções de Organismos Internacionais, Ministérios da Educação ou da Saúde, Ministério Público ou Secretarias Estaduais de Saúde ou Educação).

Pela análise dos artigos selecionados e dos demais documentos, foram propostos 40 itens de questionamento ao gestor escolar e 20 de observação direta do entrevistador. Os 60 itens foram agrupados em três dimensões, segundo sua característica principal: pedagógica, estrutural e relacional (Tabelas 1 a 5). Para cada item, está discriminado se o dado foi obtido por observação direta (observada) ou por questionamento ao gestor da escola (questionada).

A dimensão pedagógica, integrada por 14 itens, contemplou temas e atividades relacionados ao processo de aprendizagem, na perspectiva de se chegar a ambientes saudáveis. Foram considerados itens relevantes e passíveis de serem trabalhados de modo pontual ou transversal: alimentação saudável e atividade física^14^, cuidados pessoais de higiene^26^ ^,^ ^27^, saúde sexual e reprodutiva^8^; prevenção ao uso de drogas lícitas e ilícitas^2^ ^,^ ^19^ ^,^ ^22^; cultura de paz e direitos humanos e habilidades pessoais para interação, inclusão, respeito, iniciativa e tolerância^16^ ^,^ ^19^ ^,^ ^25^.

A dimensão estrutural reuniu 33 itens. Foram contemplados recursos físicos e de capacidade instalada, adequação dos espaços para as atividades pedagógicas^11^ ^,^ ^14^, e adequação da segurança pessoal e das condições sanitárias^17^ ^.^ Também apareceram itens sobre relacionamento com a comunidade do entorno da escola e parcerias que viabilizavam recursos para promoção de saúde e prevenção de doenças^13^ ^,^ ^14^.

A dimensão relacional, com 13 itens, reuniu condições consideradas necessárias na construção de um *ethos* promotor de um ambiente agradável do ponto de vista social, focando nas relações e nas condições estabelecidas na comunidade escolar. Foram contemplados aspectos sobre o relacionamento entre os alunos, professores e comunidade, a ocorrência ou não de violência, e as ações de estímulo ao protagonismo e respeito às normas de convivência^3^ ^,^ ^19^ ^,^ ^27^ ^,^ ^29^.

Cada um dos 60 itens foi redigido como pergunta, correspondendo a variáveis categóricas dicotômicas (sim ou não). Como há itens de observação direta, o uso do instrumento pressupõe a visita de quem o aplica à escola e a verificação da existência ou não da condição proposta.

A primeira versão proposta foi submetida à validação de conteúdo pelo método Delphi^10^ ^,^ ^12^ ^,^ ^24^ *.* Foram convidados cinco especialistas nas áreas de educação e saúde, contatados por meio eletrônico ou pessoalmente. Foi solicitada assinatura de termo de consentimento livre e esclarecido. Todos receberam o instrumento para opinarem se cada questão e item de orientação estavam adequadamente redigidos (sim ou não). Esse procedimento visou à compreensibilidade do questionário, e o respondente pôde apoiar suas respostas por justificativas e sugestões para melhoria do texto. Após a primeira avaliação do método Delphi, dentre as 61 questões enviadas (60 itens do instrumento e um de instrução geral), a concordância entre os avaliadores foi superior a 70,0% para todos os itens. Nenhuma questão foi desprezada e uma segunda avaliação foi realizada com apenas sete questões que receberam sugestões ou críticas. A concordância foi, então, de 100%, sem necessidade de uma terceira avaliação.

O instrumento reformulado foi apresentado aos gestores de 55 escolas de dois municípios de médio porte (cerca de 70 mil habitantes) da região Sul do Brasil. Foi solicitada às escolas a participação do diretor ou de outro membro da equipe diretiva para responder ao questionário. Cada instituição definiu quem responderia por ela. Duas escolas particulares de um mesmo município se recusaram a participar da pesquisa. Foram visitadas 31 escolas no primeiro município e 22 no segundo, totalizando 53 entrevistas. A aplicação dos questionários foi realizada por três pesquisadores após treinamento e aplicação de estudo piloto em quatro escolas de outro município com características semelhantes.

Os dados obtidos foram digitados no programa estatístico SPSS 20.0 e submetidos à análise descritiva. Nas etapas sucessivas de análise, alguns itens do questionário foram excluídos, de acordo com os seguintes critérios: questões com respostas de, no máximo, duas escolas (entre as 53 visitadas) em qualquer uma de suas categorias, pois a baixa frequência impossibilitava o pertencimento a qualquer construto; itens que, com sua exclusão, aumentavam a consistência interna da escala (ou seja, estavam baixando a consistência interna); e itens com cargas fracas (< 0,3) em mais de um fator (não pertenciam a nenhum fator).

Cada dimensão proposta pela literatura foi submetida à análise fatorial pela análise de componentes principais. A adequação dos dados para realização da amostra foi avaliada pelos testes Kaiser-Meyer-Olkin (KMO), cujo valor recomendado deve exceder 0,6, e pelo teste de esfericidade de Bartlett o qual deve atingir significância estatística (p < 0,05). O número de fatores a serem extraídos de cada dimensão foi definido utilizando-se o critério aberto, ou seja, apenas aqueles com *eigenvalues* acima de 1, a fim de identificar os fatores que estão contribuindo para a variância nas variáveis originais. Para minimizar o número de variáveis com cargas elevadas em cada fator, foi utilizada a rotação Varimax, obtendo-se os componentes representantes das dimensões subjacentes ao instrumento. Foram considerados aceitáveis valores iguais ou superiores a 0,3 para os valores das comunalidades, entendidas como as proporções das variâncias para cada variável incluída na análise que são explicadas pelos componentes extraídos.

Para assegurar que cada item correspondia à estrutura subjacente ao construto, foi considerado o critério de carga fatorial igual ou maior que 0,4 para que o item pertencesse ao mesmo. Apesar da indicação de que itens com elevada carga fatorial em mais de um fator fossem excluídos^7^, em situações onde um item contribuísse de modo mais significativo para o conjunto final de um fator, ele permaneceu ali alocado.

Para avaliar a consistência interna, utilizou-se o alpha de Cronbach, o qual avalia o quão bem um conjunto de itens mede unidimensionalmente o construto latente proposto pela escala. Consideram-se aceitáveis valores iguais ou maiores que 0,6. A confiabilidade *split-half* também foi testada. Trata-se de medida de consistência na qual a amostra é dividida em duas e os escores do teste para cada metade são comparados entre si. Se os resultados são similares, acredita-se que o mesmo construto está sendo medido nas duas metades. São aceitáveis valores mínimos de 0,6 para cada dimensão^1^.

Este estudo foi aprovado pelo Comitê de Ética em Pesquisa da Universidade do Vale do Rio dos Sinos (Processo 025/2013). Após contatos com os órgãos municipais e estaduais responsáveis pelas escolas, os respectivos gestores foram convidados a participar. Após leitura e assinatura do termo de consentimento livre e esclarecido, foram realizadas as entrevistas. No instrumento, registrou-se a identificação das escolas, sendo mantida a confidencialidade dos dados.

## RESULTADOS

Vinte e oito questões permaneceram no instrumento após as análises de validação de construto. Nas Tabelas 1, 2 e 3 são apresentados os resultados das análises de componentes principais e de consistência interna conduzidas para cada uma das dimensões propostas pela literatura.

Os requisitos exigidos para análise fatorial foram alcançados com KMO acima de 0,6 nas três dimensões e teste de esfericidade de Bartlett significativo (p < 0,001). As comunalidades da maioria dos itens também alcançaram valores desejáveis, próximos ou superiores a 0,4. Na análise de consistência interna das dimensões e fatores, a maioria das análises mostrou resultados satisfatórios para um construto homogêneo (acima de 0,6).

A Tabela 1 apresenta a dimensão pedagógica, que permaneceu com um total de 13 itens. O KMO foi igual a 0,705 e o teste de esfericidade de Bartlett alcançou significância estatística (p < 0,001). A análise de componentes principais mostrou presença de quatro componentes: drogas e sexualidade (que explicou 37,2% da variância); violência e preconceitos (16,9% da variância)*;* autocuidados (10,5% da variância); paz e qualidade de vida (9,4% da variância), todos com valores próprios superiores a 1. O valor do teste *split-half* foi de 0,856 para a dimensão. O alpha de Cronbach para os fatores extraídos foi acima de 0,7, com exceção do componente denominado paz e qualidade de vida, que teve valor de 0,61. A comunalidade (h^2^) produziu somente valores acima do mínimo recomendado de 0,3.

**Tabela 1:** Dimensão pedagógica* com itens, comunalidades e cargas fatoriais dos itens do questionário de avaliação de promoção de saúde na escola.

Tema	Item	h^2^	Fatores			
			1	2	3	4
Drogas e sexualidade						
	(Questionado) Atividades educativas na escola que estimulam o debate sobre os riscos associados ao consumo de:					
	bebidas alcoólicas	0,837	0,874	-	-	-
	tabaco (cigarros, charutos)	0,867	0,868	-	-	-
	drogas ilícitas	0,904	0,858	0,393	-	-
	(Questionado) Atividades educativas que promovam o debate sobre saúde sexual, saúde reprodutiva e DST (doenças sexualmente transmissíveis).	0,594	0,698	-	0,307	-
Violência e preconceitos						
	(Questionado) Atividades educativas de estímulo à reflexão e discussão sobre:					
	*bullying* (hostilidade, coação, constrangimento)	0,789	0,358	0,792	-	-
	discriminação e preconceito	0,784	0,382	0,751	-	-
	diversidade sexual/homofobia	0,589	-	0,728	-	-
Autocuidados						
	(Questionado) Atividades educativas sobre alimentação saudável nos diferentes espaços da escola.	0,783	-	-	0,856	-
	(Questionado) Atividades educativas relativas a habilidades pessoais como empatia, relacionamento interpessoal, tomada de decisões, pensamento crítico e criativo, manejo de tensões e/ou estresse, conhecimento de si mesmo.	0,781	-	-	0,848	-
	(Questionado) Atividades educativas que abordem e estimulem a prática da higiene corporal na escola.	0,709	-	-	0,713	0,437
Paz e qualidade de vida						
	(Questionado) Atividades educativas sobre cultura de paz e direitos humanos.	0,698	-	-	0,338	0,739
	(Questionado) Atividades educativas relativas à prática de exercícios físicos na escola, não considerando aquelas que fazem parte do currículo de Ed. Física (por exemplo, realização de jogos, gincanas, danças, lutas, corrida, ginástica, esportes coletivos ou outros).	0,551	-	-	-	0,728
	(Questionado) Atividades educativas de estímulo à reflexão e discussão sobre violências (doméstica, sexual e outras).	0,728	-	0,54	-	0,657
Alpha de Cronbach geral da dimensão	0,843	Alpha	0,89	0,74	0,79	0,61
Coeficiente de *split-half*	0,856	% Variância	37,2	16,9	10,5	9,4
KMO	0,705	% Cumulativa	37,2	54,1	64,5	73,9
Bartlett	< 0,001	-	-	-	-	-

* Os itens desta dimensão são precedidos pela orientação: Responda se a sua escola desenvolve ações/programas apresentados a seguir, de forma contínua e permanente: (Não devem ser considerados projetos em desenvolvimento, ainda não implementados, ou executados por algum período, mas já interrompidos!).

Quanto à dimensão estrutural (Tabela 2), o KMO foi igual a 0,639 e o teste Bartlett de esfericidade alcançou significância estatística (p < 0,001). Da análise de componentes principais, permaneceram três componentes: acesso, conservação e equipamentos e estrutura sanitária, com valores próprios superiores a 1, explicando, respectivamente, 33,1%, 16,1% e 14,4% da variância. O valor no teste de *split-half* para a dimensão foi de 0,805. O alpha de Cronbach foi acima de 0,7 no componente denominado *acesso,* obtendo-se valores próximos a 0,6 nos outros dois componentes (0,63 e 0,58). A comunalidade (h^2^) produziu somente valores acima do mínimo recomendado de 0,3.

**Tabela 2:** Dimensão estrutural com itens, comunalidades e cargas fatoriais dos itens do questionário de avaliação de promoção de saúde na escola.

Tema	Item	h^2^	Fatores		
			1	2	3
Acesso					
	(Questionado) A escola possui condições estruturais compatíveis com acessibilidade (ambiente físico que permita o acesso dos alunos com necessidades especiais às atividades educativas conferidas aos demais)?	0,820	0,898	-	-
	(Observado) Acessibilidade (ambiente físico que permita o acesso de pessoas portadoras de necessidades especiais a todas as atividades, como rampas, piso e salas compatíveis com a circulação de cadeiras de roda)?	0,775	0,805	-	0,334
	(Observado) Acesso ao interior da escola exclusivo por portão (ou assemelhado) monitorado por porteiro ou vigilante de modo permanente.	0,44	0,626	-	-
Conservação e equipamentos					
	(Observado) Evidência de problemas de conservação da estrutura, como presença de cadeiras quebradas em uso, buracos, goteiras, telhas quebradas, risco de quedas devido a condições do piso ou outros.	0,674	-	0,819	-
	(Observado) Biblioteca em sala exclusiva, mesas para consulta, cadeiras, estantes, proteção nas janelas com incidência de sol, e um mínimo de assentos equivalente a, no mínimo, 50,0% dos alunos da maior turma da escola.	0,658	-	0,786	-
	(Questionado) A escola possui condições estruturais compatíveis com preservação ambiental (uso sustentável de energia, plantio de árvores, reciclagem de lixo)?	0,508	-	0,505	0,414
Condições sanitárias					
	(Observado) Espaço físico/área de lazer coberta e ao ar livre em condições adequadas para atividades recreativas, não contando as áreas reservadas à prática de esportes, com área equivalente a pelo menos 1/3 da área total ocupada com salas de aula (não computar áreas de circulação).	0,698	-	-	0,818
	(Questionado) A escola possui equipe própria de saúde ou conta com o apoio de alguma equipe de serviço local de saúde que realiza avaliações periódicas de saúde e orientações a seus alunos?	0,596	-	-	0,726
	(Observado) Banheiros com condições de uso e equipamentos adequadamente preservados (vasos sanitários limpos e com água, descarga adequada, acesso a pias para higiene das mãos e limpeza geral) e escovódromo ou estruturas adequadas para escovação de dentes das crianças, incluindo as menores.	0,552	-	0,508	0,519
Alpha de Cronbach geral da dimensão	0,737	Alpha	0,72	0,63	0,58
Coeficiente de *split-half*	0,805	% Variância	33,1	16,1	14,4
KMO	0,639	% Cumulativa	33,1	49,2	63,5
Bartlett	< 0,001	-	-	-	-

Na dimensão relacional (Tabela 3), o KMO foi igual a 0,681 e o teste Bartlett de esfericidade alcançou significância estatística (p < 0,001). Na análise de componentes principais, os itens se mostraram agrupados em: relações com a comunidade (que explicou 38,1% da variância) e relações na escola (que explicou 17,1% da variância), todos com valores próprios superiores a 1. O valor no teste de *split-half* foi de 0,646 na dimensão. O alpha de Cronbach ficou abaixo de 0,7 em ambos os fatores. A comunalidade (h^2^) produziu somente valores acima do mínimo recomendado de 0,3.

**Tabela 3:** Dimensão relacional com itens, comunalidades e cargas fatoriais dos itens do questionário de avaliação de promoção de saúde na escola.

Tema	Itens	h^2^	Fatores	
			1	2
Relações com a comunidade				
	(Observado) De um modo geral, ao circular pela escola, o ambiente pode ser considerado agradável e adequado para a convivência de crianças e adolescentes.	0,634	0,794	-
	(Observado) Evidência de danos físicos à escola, como pichações, depredações ou outros indícios de vandalismo contra o patrimônio.	0,584	0,754	-
	(Questionado) A sua escola participa de organizações ou possui parcerias de interesse da população da comunidade local, envolvendo conselhos, autoridades, ONG, lideranças locais, grupos de convivência ou qualquer outro?	0,405	0,589	-
Relações na escola				
	(Questionado) Nos últimos 30 dias letivos ocorreram episódios de brigas/discussões entre pessoas da comunidade local e representantes da escola?	0,756	-	0,869
	(Questionado) Nos últimos 30 dias letivos ocorreram agressões verbais no ambiente escolar entre professores e professores?	0,529	0,402	0,606
	(Questionado) Nos últimos 30 dias letivos ocorreram agressões verbais no ambiente escolar entre alunos e professores?	0,339	-	0,613
Alpha de Cronbach geral da dimensão	0,642	Alpha	0,58	0,49
Coeficiente de *split-half*	0,646	% Variância	38,1	17,1
KMO	0,681	% Cumulativa	38,1	55,1
Bartlett	< 0,001	-	-	-

Foram excluídos 12 itens na análise de frequência das respostas (Tabela 4) e 20 nas análises de componentes principais e confiabilidade segundo os critérios de exclusão empregados (Tabela 5).

**Tabela 4:** Questões excluídas após análises de frequências, por somarem no máximo dois registros em pelo menos uma das categorias de resposta.

Dimensão	Questões
Pedagógica	Responda se a sua escola desenvolve ações/programas apresentados a seguir, de forma contínua e permanente: (Não devem ser considerados projetos em desenvolvimento, ainda não implementados, ou executados por algum período, mas já interrompidos!):
	(Questionada) Oferta na alimentação escolar e/ou venda na escola de alimentos e refeições saudáveis (por exemplo, frutas, sucos naturais, lanches ou refeições com baixo teor de açúcar, sal e gorduras)?
Estrutural	(Questionada) Os banheiros estão ligados à rede de esgotos sanitários?
	Você considera a estrutura física das salas de aula adequadas em termos de:
	(Questionada) Iluminação natural (inclui proteção contra incidência direta de raios)?
	(Questionada) Ventilação?
	(Observada) Acesso a alimentos e refeições saudáveis (por exemplo, frutas, sucos naturais, lanches ou refeições com baixo teor de açúcar, sal e gorduras).
	(Observada) Cartazes, panfletos ou qualquer outra forma de viabilização do acesso de quem circule na escola a informações sobre saúde sexual e reprodutiva.
	(Observada) Salas ventiladas, com aeração adequada e direta.
	(Observada) Iluminação adequada nas salas de aula, com localização ou proteção nas janelas contra incidência de raios solares de forma direta.
	(Observada) Mínimo de 1,20 m^2^ por aluno em cada sala (m^2^ médio sala/nº médio de alunos por sala).
Relacional	(Observada) A escola possui regras (normas definidas claramente) sobre direitos e deveres na escola?
	(Observada) Nos últimos 30 dias letivos ocorreram agressões físicas no ambiente escolar entre alunos e professores?
	(Observada) Nos últimos 30 dias letivos ocorreram agressões físicas no ambiente escolar entre professores e professores?

**Tabela 5:** Questões excluídas nos ajustes decorrentes das análises de confiabilidade ou de componentes principais.

Dimensão	Questões
Estrutural	(Questionada) Você considera que o Conselho de Pais e Mestres da sua escola está sendo um órgão efetivo?
	(Questionada) A escola desenvolve algum projeto permanente no qual os alunos tenham a oportunidade de participar de atividades educativas/recreativas fora do ambiente escolar?
	(Questionada) A escola abre seu espaço, ou oferece acesso à comunidade local para atividades educativas ou de lazer nos finais de semana? (por exemplo, escola aberta)
	Responda se a sua escola desenvolve ações/programas apresentados a seguir, de forma contínua e permanente: (Não devem ser considerados projetos em desenvolvimento, ainda não implementados, ou executados por algum período, mas já interrompidos!)
	(Questionada) Parcerias da escola com instituições/profissionais de saúde de apoio técnico na orientação sobre saúde em geral.
	(Questionada) Existem instalações adequadas para proporcionar uma alimentação saudável (refeitório próprio, com espaço e estrutura adequada)?
	(Questionada) A escola desenvolve práticas sanitárias para evitar transmissão de doenças na escola e na comunidade como o controle de vetores, como ratos e insetos e destinação adequada do lixo? (considere toda a escola).
	A escola possui condições estruturais compatíveis com:
	(Questionada) prevenção de incêndio (alvará expedido pela autoridade local)
	Você considera a estrutura física das salas de aula adequadas em termos de:
	(Questionada) prevenção de acidentes?
	(Questionada) climatização (temperatura e umidade)?
	(Observada) Acesso a alimentos com alto teor de gordura, gordura saturada, gordura trans, açúcar e sal (por exemplo, bolachas recheadas, frituras, doces em geral, salgadinhos).
	(Observada) Refeitório com estrutura adequada em termos de limpeza e organização em geral.
	(Observada) Cartazes, panfletos ou qualquer outra forma de viabilização do acesso de quem circule na escola a informações sobre tabagismo, álcool e drogas em geral.
	(Observada) Presença de câmeras de monitoramento nos espaços de circulação interna ou externa.
	(Observada) Lixeiras para destinação adequada, com separação de lixo seco e orgânico?
	(Observada) Quadra de esportes ou área própria para a prática de esportes ou atividade física, junto à escola, com espaço coberto e ao ar livre.
	(Observada) Número máximo de alunos por turma (do 5º ao 8º ano) até 35 alunos.
Relacional	(Questionada) A escola possui grêmio estudantil ou outros grupos de convivência nos quais todos os alunos têm a oportunidade de participação em processos de decisão sobre a escola?
	(Questionada) Nos últimos 30 dias letivos ocorreram agressões verbais no ambiente escolar entre alunos e alunos?
	(Questionada) Nos últimos 30 dias letivos ocorreram agressões físicas no ambiente escolar entre alunos e alunos?
	(Questionada) Nos últimos 30 dias letivos ocorreram no ambiente escolar problemas relacionados à segurança como porte de armas (brancas ou de fogo), roubo ou vandalismo, independente de terem sido acionados ou não policiais/guardas municipais/agentes de segurança?

Nenhum item da dimensão pedagógica foi excluído.

## DISCUSSÃO

O instrumento avaliado traz inovações: tem dimensão reduzida, com aplicabilidade fácil; os itens são todos apresentados na forma de questões com respostas do tipo “sim ou não”, evitando-se indefinições quanto à existência ou não daquele recurso ou processo; é o primeiro com itens de observação direta por quem faz a coleta de dados, o que reduz a influência do compromisso dos gestores com o seu ideal de escola; foi concebido para ser aplicado uma única vez em cada escola, não sendo proposto a diferentes membros da comunidade escolar, o que evita que a informação coletada reflita perspectivas ideológicas ou oscile entre insatisfação e satisfação com a escola, que pode variar largamente em uma mesma comunidade; e trabalha temas como sexualidade e violência, que fazem parte do cotidiano de comunidades em países em desenvolvimento, mas que não estão presentes ou não tem destaque em outros instrumentos.

A composição final do instrumento pode ser considerada adequada, pois, além dos valores satisfatórios na análise global de validade e confiabilidades, as dimensões estão compostas por itens que se aglutinam em componentes compatíveis com temas indicados como prioritários para a promoção de saúde no ambiente escolar. São eles: drogas^2^ ^,^ ^3^ ^,^ ^20^ ^,^ ^22^, sexualidade^4^ ^,^ ^8^ ^,^ ^13^ ^,^ ^21^, violência^19^ ^,^ ^25^, preconceitos^19^ ^,^ ^25^, autocuidados^26^ ^,^ ^27^ ^,^ ^29^, cultura de paz^19^ ^,^ ^25^ ^,^ ^27^, qualidade de vida^15^ ^,^ ^18^ ^,^ ^26^ ^,^ ^27^, acessibilidade e segurança^19^ ^,^ ^25^ ^,^ ^27^, condições sanitárias^15^ ^-^ ^18^, conservação da estrutura e equipamentos a disposição da comunidade escolar^11^ ^,^ ^14^ ^,^ ^18^ ^,^ ^27^, além das relações na escola e com a comunidade^3^ ^,^ ^4^ ^,^ ^18^ ^,^ ^24^.

Além de esforços da Organização Mundial da Saúde, que propôs um instrumento amplo para investigação de saúde na escola (o *Global School-based Student Health Survey* [GSHS])^22^, o Centro de Controle de Doenças, nos Estados Unidos, também desenvolveu o seu instrumento^26^. Iniciativas de pesquisadores em outros países também levaram à formulação de instrumentos com esta finalidade^2^ ^,^ ^16^ ^,^ ^18^. No Brasil, em 2012, a Pesquisa Nacional de Saúde do Escolar (PeNSE)^a^ incluiu em seu questionário itens sobre o ambiente escolar a serem propostos aos gestores das instituições visitadas.

Como o instrumento apresentado neste artigo, o conjunto de itens da PeNSE não empregavam escala *Likert* e havia bastante proximidade entre os dois modelos quanto à formulação de diversos itens. Não estavam contemplados itens de observação direta, já empregada em alguns estudos sobre saúde na escola, principalmente para observação de recursos estruturais, de equipamentos e área física nas dependências das escolas^19^ ^,^ ^29^.

Os instrumentos desenvolvidos até o momento^17^ ^,^ ^18^ ^,^ ^22^ ^,^ ^26^ ^,^ ^27^ compartilham a centralidade dos princípios da Carta de Ottawa: políticas de saúde na escola e habilidades pessoais em saúde, que se assemelham à dimensão pedagógica do instrumento deste estudo; ambiente físico e provisão de serviços de saúde, que se assemelham a sua dimensão estrutural; ambiente social; e relações escola e comunidade, que se assemelham à sua dimensão relacional. A origem e inserção dos pesquisadores em diferentes contextos nacionais levam os instrumentos a expressarem especificidades, que se refletem como diferenças no conjunto de seus itens. Saúde sexual, por exemplo, está presente neste e no estudo australiano^27^, enquanto o instrumento coreano^18^ analisa medidas de proteção contra desastres e situações climáticas extremas, de pouca utilidade em alguns contextos.

O questionário do GSHS tem número muito extenso de itens sobre pactuação de regras no ambiente escolar e investiga esses itens solicitando que seus termos estejam explícitos de forma escrita, o que pode não ser habitual em algumas culturas. Detalhamentos de regramentos internos expressos em documentos públicos não são comuns em escolas brasileiras, por exemplo.

Outra diferença pôde ser percebida no item segurança, já que trânsito de veículos no entorno escolar foi uma preocupação em países com menores índices de violência urbana^18^ ^,^ ^26^ ^,^ ^27^, enquanto que, para a realidade brasileira, o acesso à escola e a vigilância do ambiente escolar pareceram mais relevantes^19^. No Brasil, a violência representa um problema social e a escola está elencada como um dos espaços propícios para ajudar a enfrentar essa situação, o que requer intervenções tanto educativas quanto de segurança pública^19^ ^,^ ^25^.

A análise global da dimensão pedagógica sugeriu boa validade e confiabilidade aceitável, com maior carga fatorial no componente drogas e sexualidade. A dimensão explicita, de forma satisfatória, temas em consonância com a promoção de saúde na escola, priorizando ações que oportunizem escolhas saudáveis e autocuidado.

O tema saúde sexual apresentou boa carga fatorial, porém foi excluído na sua representação na dimensão estrutural, onde aparecia como observação direta da existência de materiais de informação sobre sexo e sexualidade nas áreas de circulação da escola. Sua exclusão se deu já na análise de frequências, pois esteve ausente em 52 das 53 escolas visitadas. Isso, aparentemente contraditório à carga do tema na dimensão pedagógica, é sugestivo da dificuldade, ainda hoje elevada, de informações relacionadas à sexualidade estarem acessíveis no contexto escolar, permanecendo como assunto evitado. Sem uma proposta mais explícita de informação, a não ser como atividade reservada à sala de aula (portanto, de cunho eventual e coletivo), o acesso a informações e algum tipo de suporte de forma individualizada, com preservação da intimidade de quem a busca, parece ter abrangência restrita^8^ ^,^ ^13^ ^,^ ^21^.

Na análise global da dimensão estrutural, foi alcançada boa validade e confiabilidade considerada aceitável. O componente relativo à estrutura sanitária apresentou alpha de Cronbach ligeiramente abaixo do considerado aceitável, porém foi composto de itens essenciais^2^ ^,^ ^11^ ^,^ ^14^ ^,^ ^26^ e sua preservação reforçava a avaliação global da dimensão. Os itens dessa dimensão apresentaram boas cargas fatoriais, com melhores resultados no componente acesso, o qual englobou aspectos relevantes em termos de recursos estruturais de acessibilidade e segurança no ambiente escolar, também em acordo com a literatura^2^ ^,^ ^16^ ^-^ ^19^ ^,^ ^27^.

Na análise geral da dimensão relacional, observou-se boa validade e confiabilidade aceitável. Foram encontradas cargas fatoriais adequadas nos itens constituintes de cada um dos componentes, que mostram o clima social da escola^19^ ^,^ ^24^. O item de participação dos alunos em processos de decisão da escola foi excluído nas análises de validade e confiabilidade deste estudo e representou uma perda de informação considerada importante^15^. Este fato pode ter decorrido do tamanho da amostra utilizada, inferior ao preconizado para análises de validação. Tem sido sugerido o número de cinco sujeitos por item^23^. Um instrumento de 60 itens deveria ser testado em cerca de 300 escolas. Talvez, aumentando o número de escolas, itens relevantes que aqui não permaneceram, seriam incluídos, ou mesmo um maior número de fatores poderia ser encontrado. Isso tem impacto, também, nas análises de confiabilidade, já que escalas com poucos itens tendem a fornecer alpha de Cronbach menores^6^.

A limitação representada pelo número de escolas onde o instrumento foi aplicado pode explicar também, pelo menos em parte, que o instrumento não tenha tido uma performance excelente, mas apenas satisfatória, com boas cargas fatoriais (> 0,4) e confiabilidade aceitável (> 0,6). O instrumento coreano^18^ foi proposto para ser respondido por membros da comunidade escolar, ao contrário deste, que tem aplicação singular. Uma coleta única por escola, por investigador adequadamente treinado e com itens de observação direta, tende a gerar dados mais objetivos. Os sete fatores daquele instrumento tiveram alphas de Cronbach variando entre 0,86 e 0,97 para o conjunto do instrumento. O instrumento coreano foi o primeiro proposto e submetido a análises de confiabilidade e de validade, inclusive com análise fatorial confirmatória. Os demais instrumentos não oferecem garantias suficientes de validade e confiabilidade.^18^ O instrumento da Austrália^2^ ^,^ ^27^, por exemplo, é referido como tendo tido bom desempenho na análise global de confiabilidade, com alpha de Cronbach de 0,88, mas sem chegar a estruturas fatoriais consistentes ao empregar a análise fatorial para validação de construto^18^.

Outra limitação deste estudo foi avaliar apenas escolas de municípios da região Sul do Brasil, semelhantes em termos de porte populacional. Estudos com outras populações e com heterogeneidade maior de ambientes escolares permitiriam avaliar a consistência dos resultados encontrados e obter melhor avaliação do instrumento.

Os dados sugerem que a estrutura proposta é coerente, com número suficiente de dimensões e fatores. O instrumento pode ser aplicado em sua forma atual, com 28 itens, mas se recomendam estudos complementares, ainda com os 60 itens originais, para que sejam incluídas realidades locais de diferentes regiões do País, com características sociais e demográficas distintas. Além disso, outras propriedades psicométricas como reprodutibilidade e validade de critério ainda necessitam ser estudadas de modo a complementar e qualificar ainda mais o processo de validação do instrumento.

## Footnotes

a Ministériodo Planejamento, Orçamento e Gestão (BR); Instituto Brasileiro de Geografia e Estatística. Pesquisa nacional de saúde do escolar 2012. Rio de Janeiro: IBGE; 2013.
